# Effects of cigarette smoking associated with sarcopenia in persons 60 years and older: a cross-sectional study in Zhejiang province

**DOI:** 10.1186/s12877-024-04993-4

**Published:** 2024-06-17

**Authors:** Junfen Lin, Meiyu Hu, Xue Gu, Tao Zhang, Haiyan Ma, Fudong Li

**Affiliations:** 1 Zhejiang Provincial Center for Disease Control and Prevention, Binsheng Road, Hangzhou, 310051 China; 2 School of Public Health, Hangzhou Normal University, Yuhangtang Road, Yuhang District, Hangzhou, 311121 Zhejiang China

**Keywords:** Sarcopenia, Elderly, Smoking, Pack-years

## Abstract

**Purpose:**

Smoking is a risk factor for sarcopenia. Nevertheless, few studies analyzed the independent effects of various smoking dimensions (duration, intensity, cumulative dose) on sarcopenia risk. This is a cross-sectional study based on an older population in Zhejiang Province to determine which smoking dimensions are mainly important for sarcopenia risk and to explore the dose–response relationship between them.

**Methods:**

Our study included 783 patients with sarcopenia and 4918 non-sarcopenic individuals. Logistic regression and restricted cubic with logistic regression (for nonlinear dose effects) were used to obtain odds ratios (ORs) and 95% confidence intervals as well as restricted cubic splines (RCS) curves.

**Results:**

Compared with never-smokers, current smokers had an increased risk of sarcopenia (OR = 1.786; 95% CI 1.387–2.301) after adjusting for confounders such as age, sex, education, alcohol consumption, disease history, etc. There was no significant association between smoking intensity and sarcopenia after more than 20 cigarettes per day (OR = 1.484; 95% CI 0.886–2.487), whereas the risk of sarcopenia increased significantly with increasing duration of smoking after more than 40 years (OR = 1.733; 95% CI 1.214–2.473). Meanwhile, there was a significant non-linear dose–response relationship between smoking duration or intensity and the risk of sarcopenia. However, the risk of sarcopenia increased linearly with the number of pack-years of smoking, which is not a significant nonlinear dose–response relationship.

**Conclusions:**

This study indicated the association between smoking and sarcopenia. Both smoking duration and cumulative dose were significantly and positively associated with sarcopenia. These findings reflect the important role of the number of years of smoking in increasing the risk of sarcopenia and provide scientific evidence that different smoking dimensions may influence the risk of the sarcopenia.

## Introduction

Sarcopenia is an age-related chronic disease, a progressive decline in total body skeletal muscle mass with low muscle strength or function [1]. Epidemiological surveys have shown that about 50 million people worldwide suffer from sarcopenia, and it is expected that the number of people suffering from the disease will be as high as 500 million by the year 2050 [2]. The Asian Working Group on Sarcopenia (AWGS) 2019 reported that the prevalence of sarcopenia in the Asian older population is 5.5% to 25.7%, which is more significant in males (5.1% to 21.0 in males and 4.1% to 16.3% in females). Nearly one-third of older adults aged 65 years and above develop sarcopenia (prevalence 14% to 33%), and the prevalence in older adults aged 80 years and older can be as high as 50% to 60% [3]. Skeletal sarcopenia will dramatically increase the risk of falls, disability, and incapacitation in older adults, and will seriously damage their health and function [4]. Therefore, early detection and intervention of risk factors for sarcopenia is important to improve the quality of older people’s life, reduce complications, and avoid serious consequences [5].

In addition to the main factors associated with the etiology of sarcopenia, studies have shown that people's lifestyles and environments are the main factors influencing the prevalence of sarcopenia [6, 7], with smoking being one of the most studied risk factors about various diseases and premature death [8]. Among older adults, smoking status has been found to be a significant risk factor for mobility impairment [9], functional status, and disability [10]. Moreover, smoking is known to cause weight and muscle mass loss as well as muscle fiber atrophy [11]. Chronic smoking causes skeletal muscle damage at the tissue or cellular level through inhibition of muscle anabolism, increased inflammatory cytokines, limitation of glucose uptake, and decreased aerobic phosphorylation capacity by toxic components of tobacco smoke [9, 12]. Therefore, chronic smoking may lead to loss of muscle mass throughout the body and result in sarcopenia. Although many previous studies have shown that history of smoking is a risk factor for developing sarcopenia [13], previous investigations on smoking and sarcopenia have not assessed the independent effects of each smoking dimension by modeling other smoking dimensions as well as exploring the dose–response relationship between smoking and sarcopenia [14]. The aim of this paper is to measure and compare the independent effects of each dimension of smoking (duration, intensity, cumulative dose); specifically, we seek to quantify the independent associations between each dimension of smoking and the risk of sarcopenia, and exploring differences in possible dose–response relationships in these associations [14]. In addition, we determine which smoking dimensions are mainly important for sarcopenia risk. This major finding may lead to relevant new information on the association between smoking and skeletal sarcopenia.

## Methods

### Data source and selection of participants

The data for this cross-sectional study is from the Zhejiang Ageing and Health Cohort Study, a community-based study that focuses on the aging and health issues of the older population in Zhejiang Province, China, implemented by the Zhejiang Provincial Center for Disease Control and Prevention. 12 counties or districts were randomly selected from 90 counties in Zhejiang Province. Then a town or street is randomly selected in each county or district. Several communities or villages were randomly selected in each town or street. All residents from the selected communities or villages who are more than 60 years old (residents must have lived in Zhejiang province for more than 6 months) are considered eligible for inclusion and are invited to participate in the study. Prior to participating in the study, participants or their legal guardians voluntarily signed an informed consent form approved by the Ethics Committee of Zhejiang Provincial Center for Disease Control and Prevention. 7 counties or districts finished baseline survey in 2014, while other 4 and 1 counties or districts finished baseline survey in 2018 and 2021 respectively.

In this study, 6,281 people had body composition measurements, grip strength as well as pace measurements by the end of 2021 in the entire cohort, and 570 were excluded because of moderate and severe depression (18), incomplete data (235) and smoking history gaps(184) or outliers (133). Finally, 5,711 participants with complete data of muscle mass, strength and physical performance by the end of 2021 were included in analysis. Data from baseline survey was analyzed in this study.

### Questionnaire

A face-to-face interview based on a self-designed questionnaire was performed by trained research assistants for each participant at the baseline survey. The questionnaire included general demographic information, family status, reproductive history, medical disease, behavioral habits, diet habits, injury, depressive symptoms, self-care ability, and cognitive function. In addition, all interviewers were trained prior to the formal survey and conducted one-on-one surveys using the tablet's face-to-face interview software.

### Measurement of each component of sarcopenia

#### Muscle mass measurement

Muscle mass was measured by bioelectrical impedance analysis (BIA) using an MC-780MA. Appendicular skeletal muscle mass (ASM) was defined as the mass of skeletal muscle in the upper and lower limbs. Skeletal muscle mass index (SMI) was calculated as ASM (kg) divided by height (m) squared(kg/m^2^).

### Muscle strength measurement

Muscle strength was assessed by grip strength, measured using a digital grip strength dynamometer. These measurements were taken in each hand, three times, and the highest value was chosen as the participant's final grip strength value.

### Measurement of physical performance

Physical performance was assessed by estimating the gait speed (GS). The participants walked along a straight path for more than 8 m at their usual speed and the gait speed was calculated for the middle 6-m course; the average of two tests was used.

### Sarcopenia classification and measurement of each component of sarcopenia

Based on the Chinese Expert Consensus on the Diagnosis and Treatment of sarcopenia in the older adults (2021), it is recommended to use the Asian Sarcopenia Working Group (AWGS) 2019 diagnostic criteria. The criteria define sarcopenia with low muscle mass and low muscle strength or performance. Low muscle mass combined with low muscle strength or low physical fitness is considered sarcopenia. Low muscle mass is defined as the ASM index < 7.0 kg/m^2^ in males and < 5.7 kg/m^2^ in females. The low strength of grip is defined as < 28 kg in males and < 18 kg in females and the low-level physical performance is defined as a gait speed < 1.0m/s.

### Definition of smoking variables

Information on smoking status, intensity, and duration was collected through the baseline questionnaire. Smoking status was categorized as never smoker, former smoker, and current smoker. Former smokers or ex-smokers were defined as subjects who had quit smoking for at least 1 year. Current smokers were defined as subjects who have smoked for a cumulative period of 6 months or more. Smoking intensity was defined as the average number of cigarettes per day and was divided into three groups ≤ 10 cigarettes, 10–20 cigarettes, and 20 or more cigarettes. The smoking duration was defined as the difference between the age at which subjects participated in the survey (current smokers and former smokers) and the age at which they started regular smoking, in other words, the total number of years smoked by former and current smokers, and was divided into four groups according to the percentile, deciles 1–3: ≤ 40 years, deciles 4–6: >40–48 years, deciles 7–8: >48–58 years and decile 9–10: > 58 years. The cumulative dose from smoking was calculated as the number of pack-year. Pack-year is a measure that takes into account the intensity and duration of smoking and is calculated by dividing the number of cigarettes smoked per day by 20 (assuming 20 cigarettes per pack) multiplied by the number of years a person smoking. We categorized smokers as light smokers (≤ 26.7 pack-years), medium smokers (> 26.7–40.5 pack-years), heavy smokers (> 40.5–55.5 pack-years), and very heavy smokers (> 55.5 pack-years) [15].

### Statistical methods

Two-tailed chi-square tests or independent t-tests were used to compare various smoking indicators and other characteristics between cases and the reference group. Unconditional binary logistic regression models were used to estimate the risk associated with smoking for sarcopenia, calculating odds ratios (or) and 95% confidence intervals (95% CI) and adjusting for other known potential confounders. All models used never smokers as the reference group. In the logistic regression models, the median of years smoked and pack-years per subgroup were used as continuous variables for trend testing. In addition, we used restricted cubic spline (RCS) logistic regression to explore the nonlinear associations of each smoking dimension with the risk of sarcopenia using the median of the smoking dimensions as a reference. The knots between 3 and 7 were tested respectively, and the model with the lowest Akaike information criterion value was selected for RCS.

## Results

### Characteristics of the participants

The sociodemographic, and various dimensions of smoking of (status, duration, pack-years) participants according to the presence of sarcopenia are summarized in Table 1. There were 784 individuals with sarcopenia in the survey population. The number of sarcopenia was similar between females and males. The mean age of study participants was 72.44 (standard deviation 6.84) years. Compared with the older people without sarcopenia, participants with sarcopenia were significantly more likely to be older, unmarried, poorly educated, retired, never worked, and minimally involved in physical exercise. However, no significant difference was observed in the test whether or not alcohol was consumed.

**Table 1:** Descriptive characteristics of participants by sarcopenia status

	No Sarcopenia	Sarcopenia	Total Sample	
Characteristic	(*n* = 4927)	(*n* = 784)	(*n* = 5711)	*P*
Age	71.62 ± 6.32	77.61 ± 7.67	72.44 ± 6.84	< 0.001
Gender				0.001
Female	2822(57.30)	399(50.90)	3221(56.40)	
Male	2105(42.70)	385(49.10)	2490(43.60)	
WHR	0.898 ± 0.066	0.873 ± 0.067	0.895 ± 0.066	< 0.001
Marital status				< 0.001
Never married	47(1.00)	7(0.90)	54(0.90)	
Married	4241(86.10)	602(76.80)	4843(84.80)	
Widowed/divorced	639(13.00)	175(22.30)	814(14.30)	
Family income				0.003
Rich	1335(27.10)	252(32.10)	1587(27.80)	
Normal	3345(67.90)	484(61.70)	3829(67.00)	
Poor	247(5.00)	48(6.10)	295(5.20)	
Education				< 0.001
Illiteracy	1885(38.30)	366(46.70)	2251(39.40)	
Primary	2360(47.90)	346(44.10)	2706(47.40)	
Junior	561(11.40)	53(6.80)	614(10.80)	
Senior and over	121(2.50)	19(2.40)	140(2.50)	
Work				< 0.001
Worked	1834(37.20)	181(23.10)	2015(35.30)	
Retired	1739(35.30)	315(40.20)	2054(36.00)	
Never worked	1354(27.50)	288(36.70)	1642(28.80)	
Alcohol				0.554
Nondrinker	3213(65.20)	500(63.80)	3713(65.00)	
Ex-drinker	180(3.70)	34(4.30)	214(3.70)	
Current drinker	1534(31.10)	250(31.90)	1784(31.20)	
Physical activity				< 0.001
Yes	915(18.60)	89(11.40)	1004(17.60)	
No	4012(81.40)	695(88.60)	4707(82.40)	
Smoke status				< 0.001
Never smoker	3696(75.00)	517(65.90)	4213(73.80)	
Current smoker	882(17.90)	207(26.40)	1089(19.10)	
Ex-smoker	349(7.10)	60(7.70)	409(7.20)	
Intensity of smoking(cigarettes/day)				< 0.001
Never smoker	3696(75.00)	517(65.90)	4213(73.80)	
≤ 10	377(7.70)	86(11.00)	463(8.10)	
10–20	717(14.60)	158(20.20)	875(15.30)	
> 20	137(2.80)	23(2.90)	160(2.80)	
Duration (years) of smoking				< 0.001
Never smoker	3696(75.00)	517(65.90)	4213(73.80)	
≤ 40	438(8.90)	48(6.10)	486(8.50)	
40–48	392(8.00)	59(7.50)	451(7.90)	
48–58	323(6.60)	93(11.90)	416(7.30)	
> 58	78(1.60)	67(8.50)	145(2.50)	
Cumulative dose (pack-years)				< 0.001
Never smoker	3696(75.00)	517(65.90)	4213(73.80)	
≤ 26.7	419(8.50)	62(7.90)	481(8.40)	
26.7–40.5	260(5.30)	62(7.90)	322(5.60)	
40.5–55.5	404(8.20)	82(10.50)	486(8.50)	
> 55.5	148(3.00)	61(7.80)	209(3.70)	

Data are presented as mean ± standard deviation or number(percent)

### Various smoking dimensions and sarcopenia

Results from binary logistic regression models are reported in Table 2. Compared with never-smokers, current smokers had an increased risk of sarcopenia (OR = 1*.*856; 95% CI = 1*.*455–2.368), controlling for age and sex; after further adjustment for various disease histories and demographic characteristics, this effect was mitigated in models 2 and 3, suggesting that the results are well robust and that the effects of other confounding factors cannot be neglected. But the increased risk for ex-smokers was not statistically significant (OR = 1.202; 95% CI = 0.834–1.733). When older people smoke 20 cigarettes or less per day, the risk of sarcopenia increases significantly with increasing smoking intensity, whereas there is no significant correlation between smoking intensity and sarcopenia after smoking intensities of more than 20 cigarettes in Table 2.

**Table 2:** Odds ratio (OR) for sarcopenia according to smoking status and intensity of smoking

		Model1		Model2		Model3	
		OR (OR 95% CI)	*P*	OR (OR 95% CI)	*P*	OR (OR 95% CI)	*P*
Smoking status							
	Never-smoker	1.00		1.00		1.00	
	Current smoker	1.856(1.455,2.368)	< 0.001	1.820(1.416,2.340)	< 0.001	1.786(1.387,2.301)	< 0.001
	Ex-smoker	1.258(0.894,1.771)	0.187	1.257(0.878,1.800)	0.212	1.202(0.834,1.733)	0.325
Intensity of smoking (cigarettes/day)							
	Never-smoker	1.00		1.00		1.00	
	≤ 10	1.517(1.114,2.064)	0.008	1.465(1.065,2.016)	0.019	1.468(1.063,2.026)	0.02
	10–20	1.828(1.409,2.373)	< 0.001	1.818(1.389,2.378)	< 0.001	1.750(1.335,2.296)	< 0.001
	> 20	1.463(0.894,2.395)	0.13	1.535(0.924,2.549)	0.098	1.484(0.886,2.487)	0.134

Model 1 was adjusted for age and gender. Model 2 was adjusted for gender, age, Waist-Hip Ratio, education, marital status, family income, work, alcohol intake, physical activity level. Model 3 was adjusted additionally for history of diabetes, hypertension, hyperlipidemia, coronary heart disease, chronic bronchitis, gallstones, arthritis, cataracts, strokes

The results in Table 3 indicated that the duration of smoking was significantly and linearly associated with the risk of developing sarcopenia (*P* < 0.001). The longer smoking duration was more strongly associated with sarcopenia than the shorter smoking duration; the risk of sarcopenia increased significantly when the smoking duration was greater than 40 years. However, there was no significant association with the risk of sarcopenia in those who had smoked for less than 40 years compared with those who had never smoked.

**Table 3:** Odds ratio (OR) for sarcopenia according to duration of smoking and cumulative dose in current and former smokers

			Model1	Model2	Model3
		Median	OR (OR 95% CI)	OR (OR 95% CI)	OR (OR 95% CI)
Duration (years) of smoking	Never- smoker	0	1.00	1.00	1.00
	≤ 40	33	1.005(0.702,1.440)	1.020(0.702,1.481)	0.976(0.669,1.425)
	40–48	45	1.703(1.208,2.399)	1.713(1.204,2.438)	1.733(1.214,2.473)
	48–58	52.5	2.027(1.502,2.737)	2.001(1.470,2.722)	1.945(1.425,2.654)
	> 58	62	2.360(1.595,3.493)	2.125(1.418,3.185)	2.057(1.365,3.100)
	^2^*P*-Trend		< 0.001	< 0.001	< 0.001
Cumulative dose (pack-years)					
	Never- smoker	0	1.00	1.00	1.00
	Light	17.5	1.156(0.828,1.614)	1.164(0.825,1.643)	1.152(0.813,1.631)
	Medium	35	1.763(1.244,2.499)	1.659(1.156,2.381)	1.599(1.107,2.308)
	Heavy	47	1.942(1.422,2.652)	1.951(1.416,2.687)	1.925(1.394,2.658)
	Very heavy	64	2.237(1.551,3.226)	2.196(1.506,3.203)	2.093(1.429,3.065)
	^2^*P*-Trend		< 0.001	< 0.001	< 0.001

Model 1 was adjusted for age and gender. Model 2 was adjusted for gender, age, Waist-Hip Ratio, education, marital status, family income, work, physical activity level, and history of diabetes. Model 3 was adjusted additionally for history of diabetes, hypertension, hyperlipidemia, coronary heart disease, chronic bronchitis, gallstones, arthritis, cataracts, strokes

^2^Test for trend based on variable containing median value for each group

Light, > 0–26.7 pack-years of smoking; Medium, > 26.7–40.5 pack-years of smoking; heavy, > 40.5–55.5 pack-years of smoking; very heavy, > 55.5 pack-years of smoking

Table 3 also shows the association between the risk of sarcopenia and pack-years by smoking level in smokers. Smokers with medium levels of exposure had a significantly increased risk of sarcopenia (OR = 1.599, 95%CI: 1.107–2.308), and the risk of sarcopenia was even higher in the heavy and very heavy smoker groups (OR = 1.925, 95%CI: 1.394–2.658; OR = 2.093, 95%CI:1.429, 3.065). Results of the three models adjusting for different confounders were consistent and stable, and all showed a general trend toward a higher risk of sarcopenia among former and current smokers with history of smoking heavier (*p*-trend < 0.001).

**Fig. 1:**
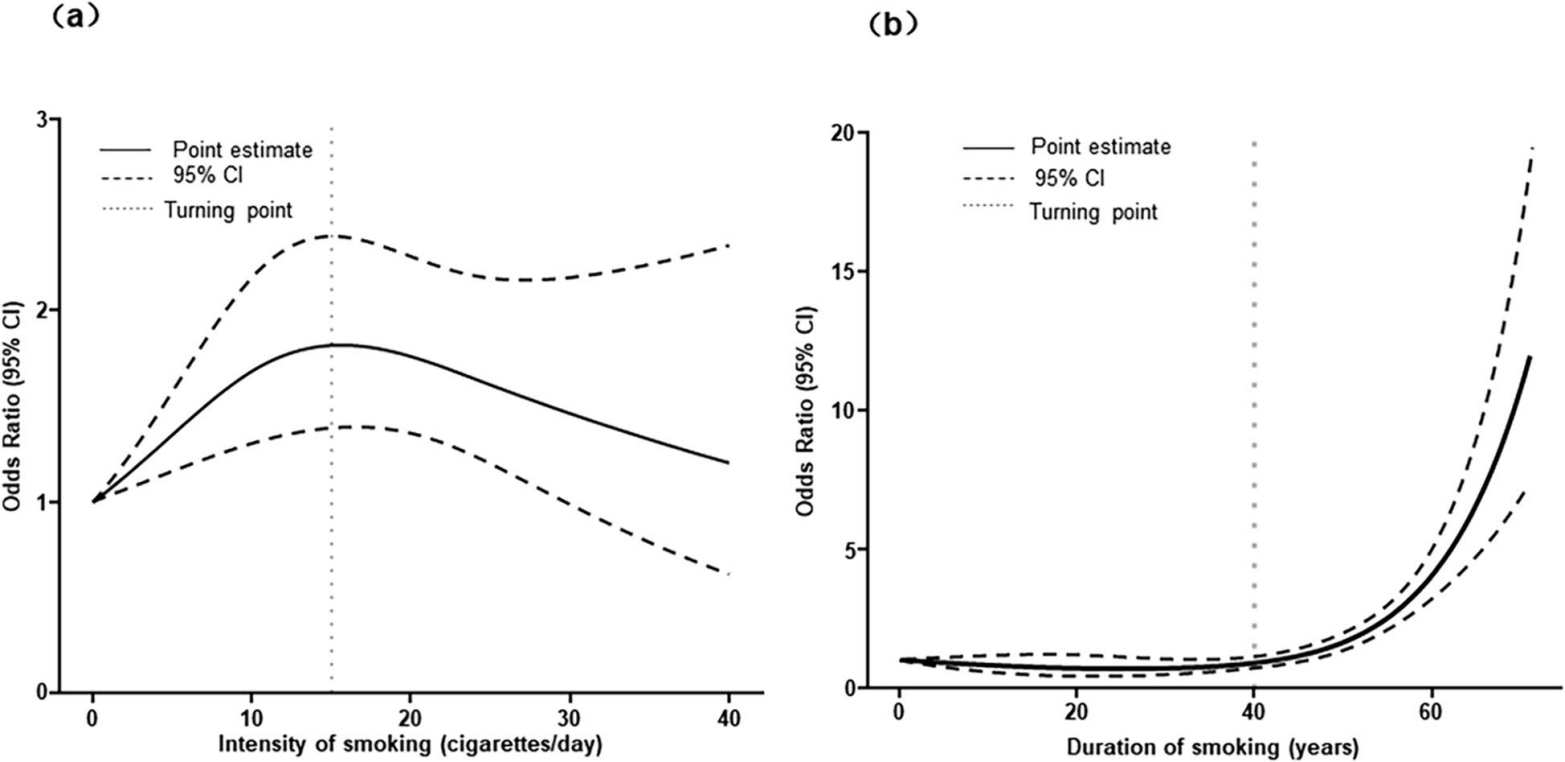
Restricted cubic spline curves for the association between intensity of smoking or duration of smoking and the risk of sarcopenia. The models were adjusted for age, gender, Waist-Hip Ratio, education level, marital status, family income, work, history of diabetes, alcohol consumption, physical activity level, and history of diabetes, hypertension, hyperlipidemia, coronary heart disease, chronic bronchitis, gallstones, arthritis, cataracts, strokes. **a** Results from the association between intensity of smoking and the risk of sarcopenia were obtained using model with 7 knots located at 2.5th, 18.33th, 34.17th, 50th, 65.83th, 81.67th, and 97.5th percentiles. **b** Results from the association between duration of smoking and the risk of sarcopenia were obtained using model with 4 knots located at 5th, 35th, 65th, and 90th percentiles

**Fig. 2:**
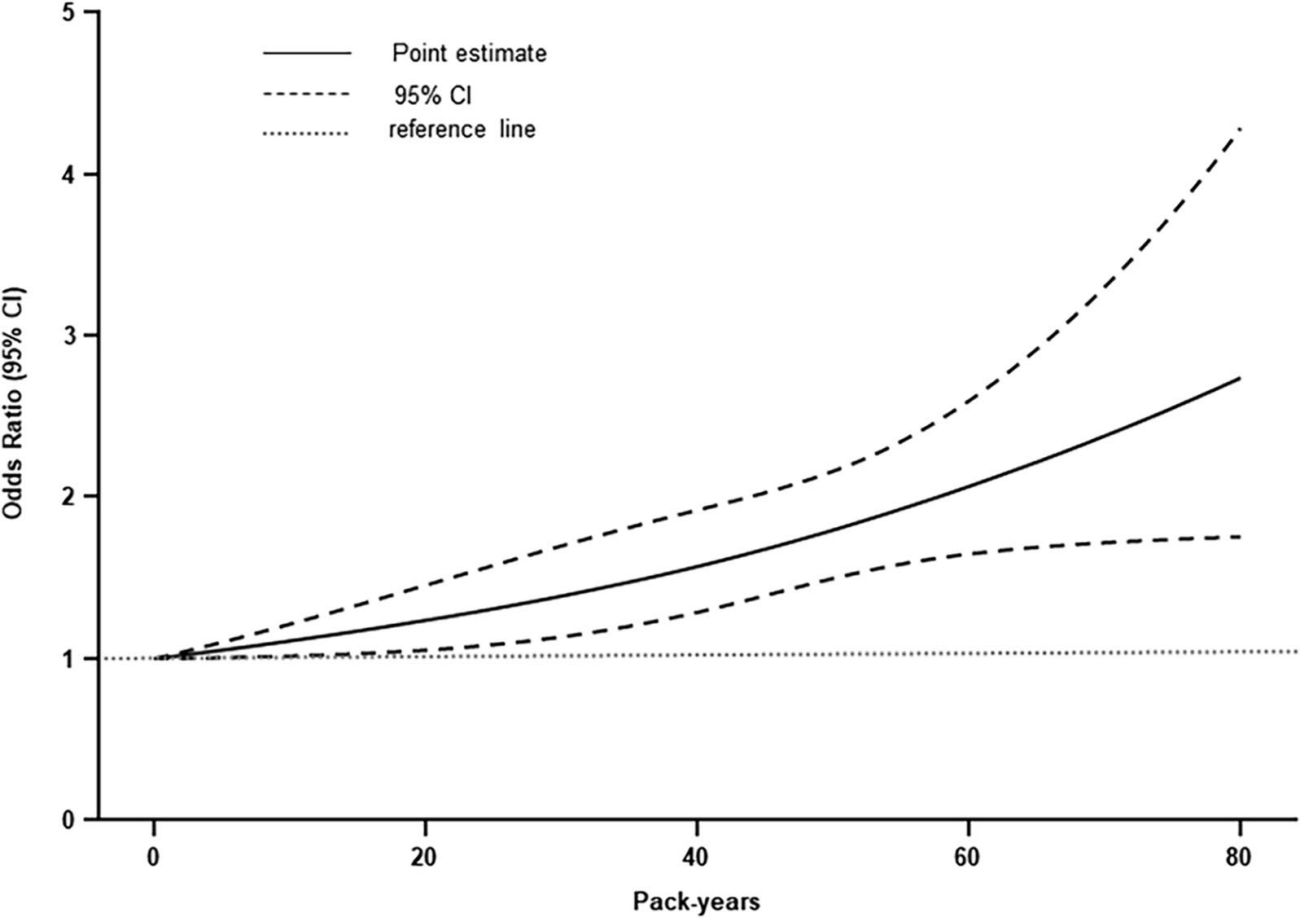
Restricted cubic spline curves for the association between pack-years and sarcopenia. After adjustment for age, gender, age, Waist-Hip Ratio, education level, marital status, family income, work, history of diabetes, alcohol consumption, physical activity level, and history of diabetes, hypertension, hyperlipidemia, coronary heart disease, chronic bronchitis, gallstones, arthritis, cataracts, strokes, results were obtained using model with 3 knots located at 10th, 50th, and 90th percentiles

Restricted cubic splines with logistic regression can better describe the changes in the risk of sarcopenia as the changes in various dimensions of smoking, as shown in Figs. 1 and 2. Figure 1a shows that after adjusting for covariates, the risk of sarcopenia increased significantly when smoking intensity below about 15 cigarettes per day on average, and then began to decline slowly as smoking intensity increased, showing a significant inverted U-shaped association (*P*_overall_ < 0.01and *P*_non-linear_ < 0.001). In Fig. 1b, we observed a relatively stable estimate of the effect before reaching 40 years of smoking, whereas the OR increased rapidly after more than 40 years of smoking, showing a J-shaped association (*P*_overall_ < 0.01 and *P*_non-linear_ < 0.001). However, in Fig. 2, the relationship between pack-years of smoking and the risk of sarcopenia does not show a significant non-linear dose–response relationship (*P*_overall_ < 0.01 and *P*_non-linear_ > 0.05).

## Discussion

Overall, the results of this study indicate that current smokers were positively associated with the development of sarcopenia compared with never-smokers, and remained statistically significant among current smokers after adjusting for potential confounders, which suggests that cigarette smoking significantly increases the risk of sarcopenia among older adults. When the smoking dimensions were modeled independently, our results further confirmed that the longer smoking duration and more cumulative smoking dose were significantly related to the increased risk of sarcopenia among former and current smokers.

Several studies have already determined that smoking is a risk factor for skeletal sarcopenia [16, 17]. The results of the present study are partially consistent with earlier findings. For example, Edward et al. [18]. found that current smoking is a reversible risk factor for skeletal sarcopenia, measuring height, weight, and muscle strength by bioelectrical impedance among community of 694 males and 1,006 females (mean 73 years) and investigating associated risk factors. Similar conclusions were obtained in another cross-sectional study, in which a significant association was found between skeletal sarcopenia and smoking in community-dwelling older Chinese men and women by recording medical illnesses, smoking and drinking habits, and using dual-energy x-ray absorptiometry to measure muscle mass and physical performance, including grip strength, timed chair stand, stride length, and timed 6-m strides in 4000 older Chinese aged 65 years [19]. In addition, a previous meta-analysis of the association between smoking and skeletal sarcopenia, which included 12 studies, showed that smoking was an independent factor contributing to the development of skeletal sarcopenia [20]. In a similar meta-analysis, Qianqian Gao et al. concluded that smoking has a negative impact on skeletal sarcopenia by pooling 29 articles addressing the relationship between skeletal sarcopenia and smoking in community-dwelling older adults [5]. However, most of the studies mentioned above only used a qualitative approach to estimate the relationship between smoking and sarcopenia instead of using a uniform assessment method of smoking status, such as the pack-year. This method was designed by the World Health Organization (WHO) in 2008 [20]. To the best of our knowledge, previous investigations have never fitted models to analyze the relationship between smoking and sarcopenia through using the pack-year and various other dimensions of smoking in China, including indicator items for ever smoking and transformed variables for continuous dimensions of smoking [14]. Therefore, we used this approach in the present study, and the results showed that pack-years of smoking was a key determinant of the risk of sarcopenia. Among ever-smokers, the risk of sarcopenia increases with the duration of smoking and pack-years increase.

In addition, we explored the nonlinear dose–effect relationship of smoking exposure on the risk of sarcopenia by using a restricted cubic spline model. Our results revealed a significant nonlinear dose–response relationship between smoking intensity or smoking duration and the risk of sarcopenia, and the shape of the RCS curves varied. The relationship between smoking intensity and the risk of sarcopenia showed the inverted U-shape, which may be due to other factors besides smoking. The development of sarcopenia is influenced not only by lifestyle including smoking, alcohol consumption, physical activity [8], and malnutrition [21], but also by changes in anabolic hormones such as oestrogen, growth hormone, and insulin-like growth factor-1 (IGF-1), inflammatory activity, and oxidative stress [22]. In addition, genetic factors may also influence the development of sarcopenia [23]. The genetic component is complex and driven by many genes [24]. A number of genes have been identified that contribute to changes in skeletal muscle mass and strength, including IGF-1 and vitamin D receptor genes [22]. These metabolic factors cannot be controlled in the model and may have an impact on the results, which may result in a U-shaped curve.

The J-shaped relationship between smoking duration and risk of sarcopenia suggests showed that the risk of sarcopenia gradually increases as the number of years of smoking increases. However, the risk of sarcopenia was not significantly non-linearly related to the cumulative dose of smoking. This indicates that the risk of sarcopenia increases linearly with cumulative smoking intensity. These are generally consistent with the findings of previously published research. An association study of smoking and loss of muscle mass and decline in muscle strength in elderly men from a Japanese community was conducted by measuring limb skeletal muscle mass and grip strength and investigating cumulative smoking exposure among 417 old men. The results found that ASM index and grip strength progressively decreased with increasing levels of cumulative smoking exposure (pack-years), suggesting that smoking leads to loss of muscle mass and function in older men [25], thereby increasing the risk of sarcopenia. M. Locquet et al. [26] conducted a cohort study assessing the association between smoking status or number of cigarettes smoked per day and the incidence of sarcopenia or severe sarcopenia during a 5-year follow-up period, which found that smokers had a 2.36-fold higher risk of sarcopenia than non-smokers, and smokers had a 2.68-fold higher risk of severe sarcopenia than non-smokers (95% CI: 1.21 -5.93). This further confirms that increased levels of smoking correspondingly increase the risk of sarcopenia.

Several studies have reported some possible mechanisms of sarcopenia caused by smoking. The effects of smoking on skeletal muscle structure and metabolism are now confirmed in clinical, in vivo and in vitro research. Firstly, tobacco smoke is a complex aerosol containing thousands of harmful substances such as nicotine, polycyclic aromatic hydrocarbons, carbon monoxide, nitrogen oxide compounds, aldehydes, etc. [27]. Acetaldehyde can give rise to muscle fiber damage, and acrolein can cause muscle atrophy and degradation of myosin in mice [28]. These tobacco smoke gas-phase components enter the circulatory system and reach the skeletal muscle tissues, which may affect their metabolism and protein disorders, leading to muscle damage [20]. So long-term smoking may result in weight and muscle mass loss as well as muscle fiber atrophy [11, 29]. Secondly, it was shown in vivo experiments that tobacco smoke can induce skeletal muscle damage mediated by inflammation and muscle oxidative stress [30]. Moreover, smoking is a causative factor for sarcopenia. Several vivo and vitro studies have shown that smoking can contribute to skeletal muscle damage by disrupting skeletal muscle metabolism, increasing skeletal muscle inflammation, and oxidative stress, as well as the overexpression of genes associated with muscle wasting and activating various intracellular signaling pathways [31]. In addition, smoking can also affect the expression levels of myosin and proteins associated with catabolism. Petersen et al. found that the synthesis of some muscle proteins had decreased and expression of muscle atrophy-related genes and myogenesis inhibitory proteins increased among smokers, compared with non-smokers [32]. Myogenesis inhibitors are members of the tumor growth factors that inhibit skeletal myogenesis and negatively regulate muscle mass. Therefore, smoking can increase the risk of sarcopenia by impairing muscle protein metabolism and increasing the expression of genes associated with disruption of muscle protein homeostasis [31].

We should acknowledge some limitations of the present study. Firstly, there may be recall bias in asking for information about the history of smoking from participants' memories. Secondly, causality could not be confirmed due to the cross-sectional study design. Thirdly, although this study attempted to adjust for confounders, the effects of other potential confounders such as stress, sleep, mood, and passive smoking could not be completely excluded. Finally, our study lacked detailed information on smokers who quit smoking, and further research is needed to investigate the relationship between smoking cessation and the risk of sarcopenia in the future. The main strengths of our study include the fact that this is a cross-sectional study of sarcopenia in Zhejiang Province with a larger sample which represents the majority of the older adults in Zhejiang so far, thus improving accuracy. Furthermore, our analytical methods not only analyzed the qualitative effects of smoking but also quantitatively assessed the effects of each smoking dimension on sarcopenia.

## Conclusions

In conclusion, our study confirms the positive association between smoking and the risk of sarcopenia and further validates the relationship by independently modeling the dimensions of smoking. We found a significant non-linear dose–response relationship between smoking intensity or duration of smoking and the risk of sarcopenia, and that the risk of sarcopenia decreased after more than 15 cigarettes per day and was no longer significantly associated with smoking intensity after more than 20 cigarettes per day. Moreover, the risk of sarcopenia significantly increased after more than 40 years with smoking duration increased. These results reflect the important influence of longer smoking duration on sarcopenia. In addition, the cumulative dose of smoking was not significantly associated with the light group, and the risk of sarcopenia increased linearly after reaching medium smoking. The above findings show that it is valuable to reduce the number of years of smoking, even for smokers with high smoking intensity. These research conclusions may have implications for the prevention of sarcopenia or provide scientific clues to gain insight into the mechanisms of smoking-induced sarcopenia.

## Data Availability

The datasets generated and/or analyzed during the current study are not publicly available due to legal restrictions but are available from the corresponding author on reasonable request.

## Abbreviations

RCS: Restricted cubic spline
OR: Odds ratios
BIA: Bioelectrical impedance analysis
ASM: Appendicular skeletal muscle mass
SMI: Skeletal muscle mass index
GS: Gait speed
AWGS: Asian Sarcopenia Working Group
